# Recurrence of gigantic overhanging bleb post excision: a case report

**DOI:** 10.3205/oc000224

**Published:** 2023-09-19

**Authors:** Kirti Singh, Mainak Bhattacharyya, Ravinder Saran, Nikhil Gotmare, Himshikha Aggarwal, Pragya Jain

**Affiliations:** 1 Glaucoma Division, Guru Nanak Eye Centre, New Delhi, India; 2 Pathology Department, Gobind Ballabh Pant Institute of Post Graduate Medical Education & Research, New Delhi, India

**Keywords:** amniotic membrane, bleb, overhanging, recurrence, trabeculectomy

## Abstract

**Purpose:**

To report the case of an extremely large overhanging bleb, extending from superior fornix to limbus, in a 57-year-old poorly controlled diabetic, six years after trabeculectomy for an uncontrolled primary open angle glaucoma (POAG) with recurrence, months after complete excision.

**Methods:**

An overhanging bleb is defined as a filtering cicatrix which has been massaged downward over the cornea by eyelid action. It has been linked to anti-metabolite use during glaucoma filtering surgery. Despite being functional, these blebs result in patient discomfort ranging from foreign body sensation and lacrimation to dysphotopsia. A 57-year-old male presented with complaints of reduced vision, foreign body sensation, watering, and difficulty in eye closure in the left eye (OS) for past 6 months. He had undergone trabeculectomy with mitomycin C 6 years ago for advanced primary open-angle glaucoma with no follow-up beyond the initial one 4 weeks post-surgery.

**Results:**

At presentation, he had a giant multi-loculated, cystic filtering bleb (15 mm x 8 mm x 4–5 mm), which was carefully excised. Amniotic membrane was used as an anti-fibrotic as well to cover the defect. Seven months after surgery, there was recurrence of this overhanging cystic bleb when it was again excised with debulking of the conjunctiva done and cryotherapy applied to its margins.

**Conclusion:**

Although multiloculated cystic overhanging blebs have been documented before, such a large (posterior extent till fornix), thick-walled multiloculated bleb with histopathological evidence of chronic inflammatory process has not been reported prior.

## Introduction

Overhanging blebs, often called over-filtering blebs, have been linked to injudicious antimetabolite use during glaucoma filtering surgery [1], [2], [3]. Despite being functional, these blebs result in patient discomfort ranging from foreign body sensation and lacrimation to dysphotopsia and impaired vision due to induced astigmatism or visual axis occlusion and tear film instability [3]. The cosmetic blemish of these unsightly blebs coupled with the constant threat of impending infection or rupture warrants bleb revision in most cases [2].

Revision by surgical and non-surgical means seeks to de-bulk the bleb without compromising its function. The non-surgical methods of revision include use of autologous blood injection, cryo or neodymium-doped yttrium aluminum garnet (Nd:YAG) laser application [4], [5]. Surgical approaches include compression sutures, bleb window cryopexy, and bleb-limiting conjunctivoplasty [2], [4], [5], [6]. Recurrence of overhanging bleb post-bleb excision is extremely rare. We present the case of an extremely large overhanging bleb, extending from superior fornix to limbus, with recurrence months after complete excision. 

## Case description

A 57-year-old male presented with complaints of reduced vision, foreign body sensation, watering, and difficulty in eye closure in the left eye (OS) for the past 6 months. He had undergone trabeculectomy with mitomycin C (MMC) application (0.02% for 3 min) 6 years ago for advanced primary open-angle glaucoma at our tertiary eye hospital. 

The post-operative recovery of the patient had been unsupervised beyond 4 weeks as he left for an overseas job and used steroid (prednisolone acetate 1%) and lubricant (CMC 0.5%) eye drops on and off without consultation and any documentation for 2–3 months. Around 11 months prior to his presentation at our hospital, he had consulted a local ophthalmologist and had been prescribed 2 anti-glaucoma medications (travoprost 0.004% and dorzox 2%) for both eyes (OU). The patient was a diabetic with poor glycemic control on oral hypoglycemic drugs.

**Figure 1:**
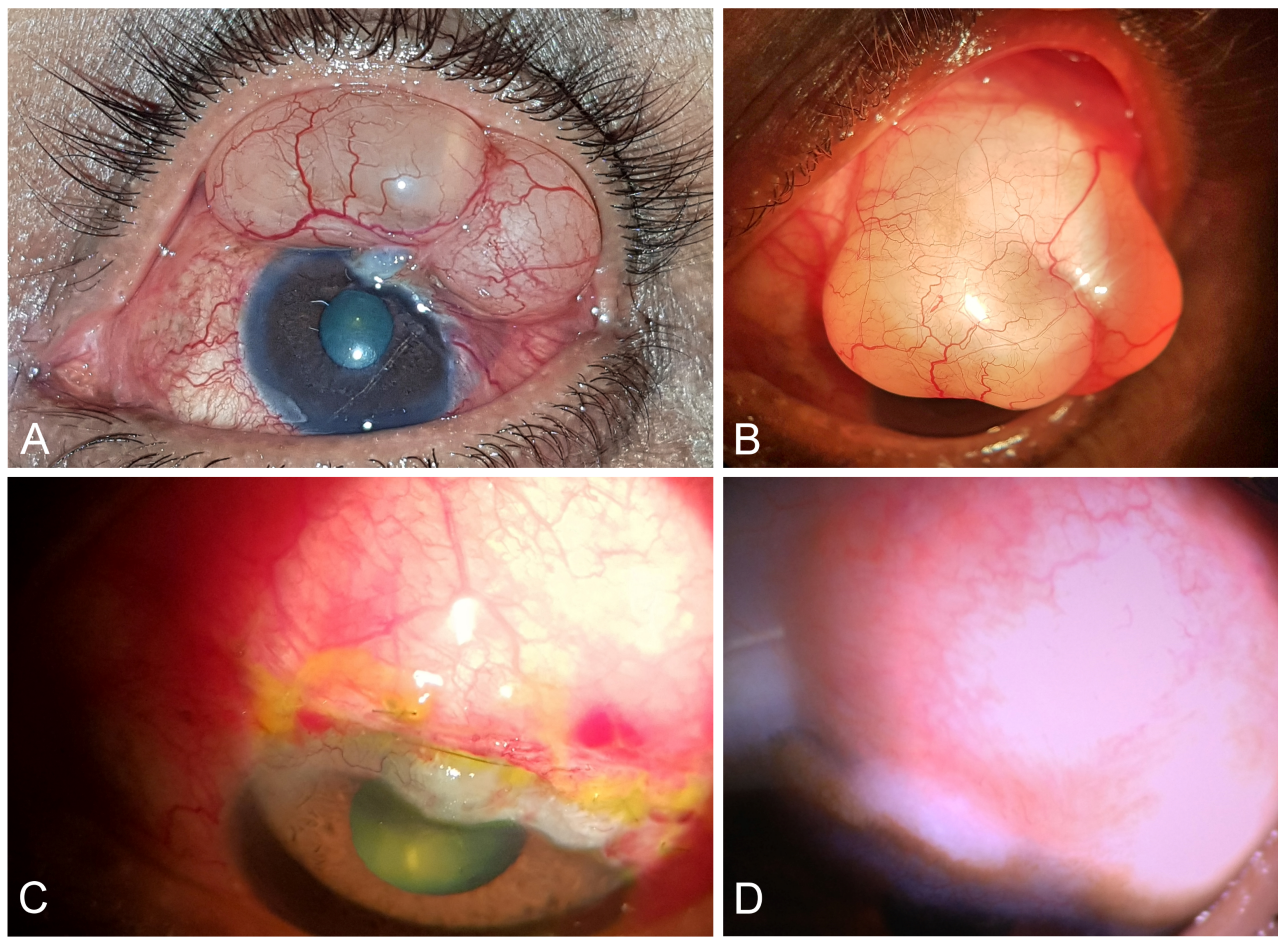
Bleb morphology A: Multi-loculated bleb extending 5 clock hours, B: High bleb extending posteriorly up to fornix, C: Post-excision bleb appearance on day 14, D: Post-op bleb after suture removal

On examination a large filtering bleb extending horizontally for 5–6 clock hours and overhanging vertically on the superior cornea was seen (Figure 1A (Fig. 1)). The bleb dimensions were 15 mm x 8 mm (H x V) with a height of 4–5 mm. Morphological appearance was thick walled, multi-loculated, and cystic, with the posterior extent almost up to the superior fornix (Figure 1B (Fig. 1)). 

The patient’s vision had deteriorated from 6/36 (20/120) at the time of trabeculectomy to 2/60 (20/502) at present. He had also developed grade 2 immature senile cataract OS; fundus examination, albeit difficult due to media haze, revealed a cup-disc ratio of 0.8:1 but no signs of chorio-retinal folds or disc edema. His intraocular pressure (IOP) at presentation was 18–20 mmHg in the left eye on 2 drugs. Perimetry was not possible due to poor visual acuity (VA) (2/60), but previous visual field (VF) (prior to trabeculectomy) showed biarcuate scotoma in left eye. His right eye also showed moderate glaucomatous damage (CDR 0.6:1) (highest IOP (as per previous records)=30 mmHg) with VA 6/9 (20/30) but was medically controlled presently.

### Surgical technique for primary excision

The patient was taken up for bleb excision after informed consent. The surgical technique involved blunt dissection of the corneal aspect of the bleb, continued posteriorly. The extensive sub-conjunctival fibrotic adhesions were lysed. No underlying scleral thinning or necrosis was seen. The redundant lax conjunctiva was excised and the bleb reformed with viable conjunctiva being approximated to limbus with 8–0 nylon. Amniotic membrane was placed in sub-conjunctival space to act as an anti-fibrotic. The newly reconstructed bleb was titrated at the end of surgery and no fluid leak was noted. Post-operatively the bleb was shallow with angry vasculature, which resolved to mature into a diffuse bleb (IBAGS grading H2E3V2S0) by day 14 (Figure 1C and D (Fig. 1)).

**Figure 2:**
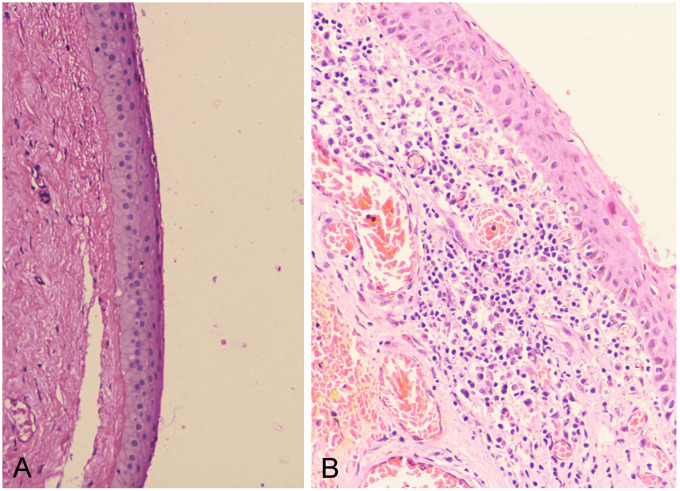
A: Section showed corneal epithelium with slit (artifact). The deeper tissue is dense sclerotic (X 4 HE). B: Focal dense lymphoplasmacytic cells collection noted (X 20 HE)

Histopathology of excised bleb tissue revealed a lining of stratified squamous epithelium with sub-epithelial connective tissue showing focal chronic inflammatory cells, collection of lymphoplasmacytic cells, congested vessels and occasional goblet cells suggestive of conjunctival cyst (Figure 2 (Fig. 2)).

**Figure 3:**
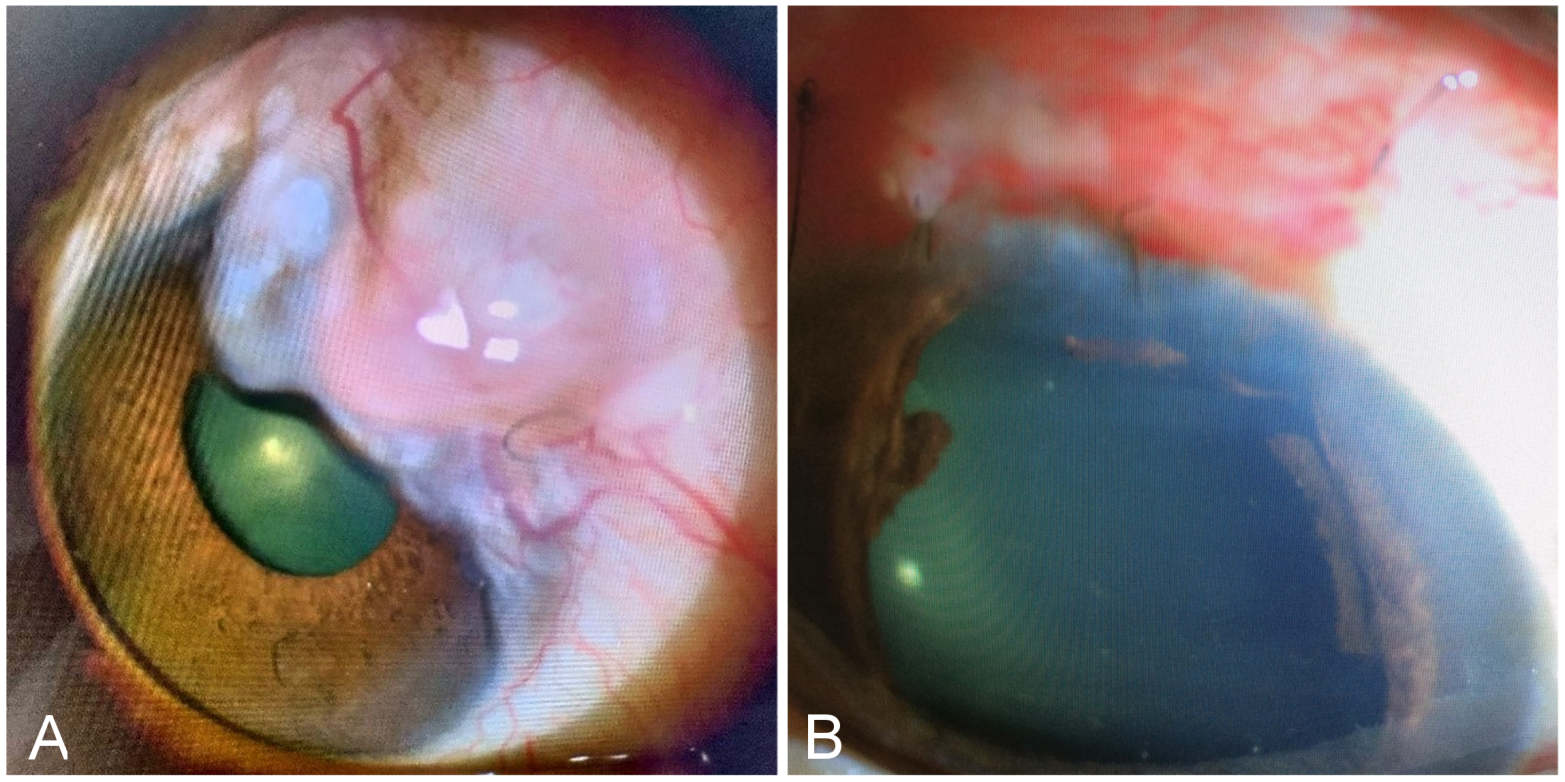
A: recurrence of overhanging bleb post excision, B: post-re-excision bleb appearance

Post-operative recovery was uneventful with visual acuity of 6/60 (20/200) and IOP ranging from 14 to 16 mmHg without requirement of any anti-glaucoma medication. Seven months after surgery, the extent of the bleb started to expand again, with dimensions of H3E4V2S0. A single cystic swelling was seen extending from 1 to 2 o’clock (Figure 3A (Fig. 3)). The patient was planned for a repeat excision combined with the extirpation of the inner bleb wall. 

### Surgical technique for re-excision

Debulking of the overhanging conjunctiva was done followed by cryotherapy with liquid nitrogen performed using 1.5 mm cryoprobe at the margins of the remnant conjunctiva to induce scarring. Iris prolapse was noted intra-operatively and the iris had to be partially abscissed and reposited after enlarging the previous surgical peripheral iridotomy (PI). The bleb was formed on table and no leak was noted.

At the 6-month follow-up following the re-excision, the patient was stable with no visual deterioration and well-controlled IOP (12–14 mmHg) without the need for any anti-glaucoma medications (Figure 3B (Fig. 3)).

## Discussion

An overhanging bleb is defined as a filtering cicatrix which has been massaged downward over the cornea by eyelid action [1]. The explanation given for this variation of bleb healing is aqueous overflow dissecting a path between corneal epithelium and stroma. A report by Ito et al. documents a double bleb separated by a clear membrane with both being functional [7]. Deranged wound healing implicating inflammatory cells and markers has been linked to overhanging blebs. Myofibroblasts migration has been documented by immunohistochemistry studies [8]. Healing propensity linked to inflammatory markers has been identified in a study documenting intense activity of TGF-β2 (pro-fibrotic) coupled with reduction of COL1A1 (anti-scarring protein) [3]. 

Most overhanging blebs lie on the surface of Bowman’s layer and can be easily dissected off the underlying cornea. Anterior segment imaging by anterior segment optical coherence tomography (AS-OCT) or ultrasound bio-microscopy helps in delineating the depth and extent of the bleb overhanging over the cornea and guides in surgical decisions regarding the requirement for donor corneal tissue for bridging the defect. 

No age group is exempt (reported for 12–82 years of age) and it has been known to occur days to years after the filtering procedure [2], [5]. Propensity for the formation of overhanging blebs has been linked to liberal use of antimetabolites; therefore we used a more physiological antifibrotic agent, namely amniotic membrane instead of MMC [2], [9]. Liquid nitrogen cryotherapy induces scarring of the conjunctiva and creates adhesion between the superficial conjunctiva and underlying Tenon capsule and sclera and was hence used during re-excision.

Although multiloculated cystic overhanging blebs have been documented before, such large, multiloculated blebs with thick walls and posterior extent up to the fornix have not been reported [10]. The cystic transformation of a bleb over a 6-year period, with recurrence within 7 months of bleb excision, gives credence to bleb hypertrophy subsequent to persistent inflammation. Poor metabolic control of diabetes coupled with an inherent propensity for uncontrolled healing could be contributory. 

## Notes

### Patient consent

The authors certify that they have obtained all appropriate patient consent forms in which the patient has given his consent for his images and other clinical information to be reported in the journal. The patient was assured that his name and initials will not be published and due efforts would be made to conceal his identity, though anonymity cannot be guaranteed.

### Competing interests

The authors declare that they have no competing interests.
